# Estimating age conditional probability of developing disease from surveillance data

**DOI:** 10.1186/1478-7954-2-6

**Published:** 2004-07-27

**Authors:** Michael P Fay

**Affiliations:** 1 National Cancer Institute 6116 Executive Blvd., Suite 504 Bethesda, MD 20892-8317, USA; 2 (Current Address) National Institute of Allergy and Infectious Diseases, 6700 B Rockledge Drive MSC 7609, Bethesda, MD 20892-7609, USA

## Abstract

Fay, Pfeiffer, Cronin, Le, and Feuer (*Statistics in Medicine *2003; **22; **1837–1848) developed a formula to calculate the age-conditional probability of developing a disease for the first time (ACPDvD) for a hypothetical cohort. The novelty of the formula of Fay et al (2003) is that one need not know the rates of first incidence of disease per person-years alive *and disease-free*, but may input the rates of first incidence per person-years alive only. Similarly the formula uses rates of death from disease and death from other causes per person-years alive. The rates per person-years alive are much easier to estimate than per person-years alive and disease-free. Fay et al (2003) used simple piecewise constant models for all three rate functions which have constant rates within each age group. In this paper, we detail a method for estimating rate functions which does not have jumps at the beginning of age groupings, and need not be constant within age groupings. We call this method the mid-age group joinpoint (MAJ) model for the rates. The drawback of the MAJ model is that numerical integration must be used to estimate the resulting ACPDvD. To increase computational speed, we offer a piecewise approximation to the MAJ model, which we call the piecewise mid-age group joinpoint (PMAJ) model. The PMAJ model for the rates input into the formula for ACPDvD described in Fay et al (2003) is the current method used in the freely available DevCan software made available by the National Cancer Institute.

## Background

Fay, Pfeiffer, Cronin, Le, and Feuer [1] showed how to calculate the age-conditional probabilities of developing a disease (ACPDvD) from registry data. Throughout this paper we use "cancer" as our disease of interest, but the method applies to specific types of cancer as well as other diseases where information is collected by population based surveillance methods. Fay et al [1] provided a formula (see equation 1 below) to calculate ACPDvD after inputing the rate function by age of (1) first incidence of cancer per person-years alive, (2) death from cancer per person-years alive, and (3) death from other causes per person-years alive. Fay et al [1] used a simple piecewise constant model for the three rate functions, which have constant rates within each age group.

**Figure 1:**
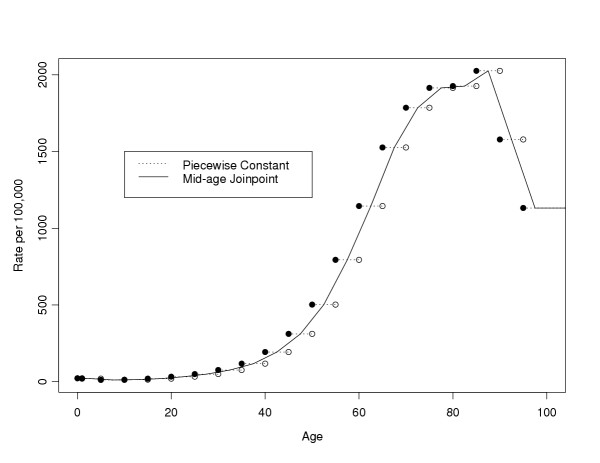
SEER 12 all invasive cancer incidence rates, 1998–2000, all races, both sexes: Piecewise constant and mid-age joinpoint methods.

Here we detail two more complicated models for the rates. The first model is a segmented regression model or joinpoint model for the rates, where the rate function is a series of linear functions that join at the mid-points of the age groups, and the rate function is constant before the first mid-point and after the last "mid-point" (because the last interval goes to infinity, the last "mid-point" is not really a mid-point at all, see below). We will call this model the MAJ (mid-age group joinpoint) model for the rates. In Figure 1 we show how both the piecewise constant model and the mid-age group joinpoint model apply to all invasive cancer incidence from the Surveillance Epidemiology and End Results (SEER) program of the U.S. National Cancer Institute in 1998–2000. Figure 1 uses the SEER 12 registries which cover about 14 percent of the U.S. population, covering 5 states (Connecticut, Hawaii, Iowa, New Mexico, Utah), 6 metropolitan areas (Atlanta, Detroit, Los Angeles, San Francisco-Oakland, San Jose-Monterey, Seattle-Puget Sound) and the Alaska Native Registry (see [2]). Similar graphs showing the MAJ model can be made for the other rates required in the calculations, death from cancer and death from other causes per person-years alive.

Notice that the MAJ model gives a more smoothly changing and probably a better modeled rate. The only place where the MAJ model may not perform better than the piecewise constant model is at peaks or valleys, where there may be some bias. In Figure 1 we see that the smoothness of the MAJ appears to produce more plausible estimates for ages 0 through 85 and from ages 90 and above, and the only age group with a noteworthy bias problem is 85 to 90. Thus, for almost all of the age range the MAJ model is more plausible.

A problem with the mid-age group joinpoint model is that it requires numeric integration for its calculation. The second model uses a series of piecewise constant values to approximate the mid-age group joinpoint model. We call this second model the PMAJ (piecewise mid-age group joinpoint) model. The PMAJ does not require numeric integration, so it is much faster than the MAJ model. The PMAJ model is a piecewise constant model that only differs from the piecewise constant model of Fay et al [1] in that the pieces are smaller and the corresponding values of the rates are motivated by the MAJ model. Starting with version 5.0, the freely available DevCan software [3] uses the PMAJ method. (There was a small calculation error in versions 5.0 and 5.1 that has been corrected in version 5.2). DevCan calculates ACPDvD or age conditional probability of dying from a disease for U.S. cancer data or for user supplied data.

The outline of this paper is as follows. The review and overview section reviews the issues in estimating the age conditional probability of developing disease from surveillance data. This section includes a motivation for using this type of statistic to describe population data. The review and overview section additionally gives graphical descriptions of the MAJ and PMAJ methods. The paper is structured so that readers not interested in the details may skip the next two sections and the appendix, which give precise and notationally involved definitions of the MAJ estimators. The examples and discussion section gives examples of the estimator of ACPDvD using three different methods for estimating the rates, the simple piecewise constant method proposed in Fay et al [1], the MAJ method, and the PMAJ method. In supplimental material [see Additional file 1] we compare the PMAJ method with the method of Wun, et al [4], since the latter method was the method used in versions of the DevCan software before version 5.0.

## Review and overview

Consider a surveillance program like the SEER program of the U.S. National Cancer Institute. This program attempts to count every incidence of cancer within the catchment area of the program. Because cancer is a disease in which the rates of the disease are highly dependent on age, in order to give interpretability to the counts within the SEER registries, we must somehow account for the age distribution in the popoulation.

One simple and popular statistic is the age adjusted rate or directly standardized rate (DSR). In the SEER Cancer Statistics Review [2] DSRs are used to compare different cancer sites, trends on specific cancer sites over time, and rates by sex and race. The DSR is calculated by a simple weighted sum of the age specific rates for each 5 year age group, where the weights are proportional to the U.S. 2000 population. Thus, the DSR may be interpreted as the rates adjusted as if all the populations being compared had age distributions similar to the U.S. 2000 population. The DSRs are useful for gaining an overall picture of how the incidence and mortality of each cancer effects different populations (e.g., different races, SEER population at different times), while controling for the effect of differing age distributions between populations being compared. A disadvantage of the DSR is that it is hard to relate to an individual's risk. For example, Table I-4 of the SEER Cancer Statistics Review, 1975–2000 [2] states that the DSR for breast cancer for females for the years 1996–2000 is 135 per 100,000 person-years. The average American woman may wonder, how does that relate to my risk? Will I be likely to get breast cancer in my lifetime? If I am 40 years old now, what is my risk of getting breast cancer in the next 10 years given that I have survived to this old without getting it? These questions are the motivation for using the age conditional probability of developing disease (ACPDvD), and in order to estimate the ACPDvD for female breast cancer, we require information not only about the rate of female breast cancer but also about the rates of dying from female breast cancer and dying from other causes.

The ACPDvD uses cross-sectional incidence and mortality rates to estimate the age-conditional probabilities of developing disease in a hypothetical cohort in which we assume the age specific rates do not change over time. This gives a personal interpretation to the cross-sectional data, allowing statements like the following: if the incidence and mortality rates remain at their present values (as observed in SEER 12, 1998–2000), then a female born today would have a 13.5% chance of developing breast cancer over her lifetime (see Table 2). We can also calculate ACPDvD over intervals. For example, a female who has reached 40 years old without developing breast cancer has a 1.5% chance of developing breast cancer by the time she is 50.

**Table 2:** Age Conditional Probability of Developing Different Types of Invasive Cancers (in Percent) from SEER 12, 1998–2000

Start Age	End Age	Model	All Invasive (Both Sexes)	Prostat(Male)	Breast (Female)	Acute Lymphocytic Leukemia (Both Sexes)
0	20	Piecewise const	0.3158	0.0009	0.0015	0.0669
		PMAJ, interval = .5	0.3260	0.0011	0.0021	0.0633
		MAJ	0.3260	0.0011	0.0021	0.0633
0	50	Piecewise const	4.0690	0.2002	1.9188	0.0837
		PMAJ, interval = .5	4.1657	0.2550	1.9492	0.0808
		MAJ	4.1657	0.2550	1.9492	0.0808
40	50	Piecewise const	2.5260	0.2032	1.5131	0.0053
		PMAJ, interval = .5	2.5976	0.2579	1.5169	0.0055
		MAJ	2.5975	0.2579	1.5169	0.0055
0	Inf	Piecewise const	42.0876	17.4952	13.6471	0.1154
		PMAJ, interval = .5	41.7547	17.3375	13.5477	0.1121
		MAJ	41.7574	17.3389	13.5485	0.1121
60	61	Piecewise const	1.2340	0.5989	0.3822	0.0009
		PMAJ, interval = .5	1.0852	0.4946	0.3627	0.0009
		MAJ	1.0852	0.4946	0.3627	0.0009
64	65	Piecewise const	1.2758	0.6131	0.3872	0.0009
		PMAJ, interval = .5	1.4453	0.7440	0.4045	0.0010
		MAJ	1.4453	0.7440	0.4045	0.0010
60	65	Piecewise const	6.0331	2.9128	1.8777	0.0042
		PMAJ, interval = .5	6.0622	2.9492	1.8758	0.0044
		MAJ	6.0622	2.9492	1.8759	0.0044

Calculation of the ACPDvD is somewhat complicated, and we describe the complications in relation to the simple DSRs. Consider first the age specific incidence rates which are used to calculate the DSRs. These rates simply count the number of incident cases of a particular disease (e.g., female breast cancer) within each age group and divide by the total number of person-years estimated by the population. For counts of a single year, the person-years are estimated by the mid-year population of the catchment area (for sex-specific cancers like prostate cancer or female breast cancer, we only use the population of the appropriate sex). Note that the incident cases may include individuals who have previously been diagnosed with the cancer and have developed a new primary cancer.

For the ACPDvD for any specific disease we would like the rate of first incidence per person-years alive and disease-free. Thus, there are two difficulties, (1) the usual age specific incidence rates include persons with multiple primary cancers, and (2) the denominators include persons who have previously been diagnosed. Merrill and Feuer [5] discuss both difficulties and adjust for them creating risk-adjusted cancer incidence rates. Merrill and Feuer [5] study the effect of these adjustments for several cancer sites. To handle the first difficulty, (similar to [5]) we can remove cases where we have a record of a previous diagnosis of that particular type of cancer. Because the registries in SEER were not all begun at the same time, to avoid bias the DevCan program only searches the records for previous cancers back until the year when the last registry was added. This year is denoted the follow-back year. (If the disease of interest is any malignant cancer, then the difficulty is handled differently. Although at each cancer record we do not record what specific types of cancers were previously diagnosed for the person, we do know whether any tumors were previously diagnosed. Thus, if the disease of interest is any malignant cancer and if the record states there was a previously diagnosed tumor, then we assume that the previously diagnosed tumor was malignant, and do not count that case as a first incidence.) To handle the second difficulty, the additional person-years in the denominator, Merrill and Feuer [5] adjust the denominator by multiplying the age-specific population by 1 minus an estimate of the prevalence of the disease in the population. Merrill and Feuer [5] also estimate the prevalence of medical procedures which remove individuals from the at-risk population, such as hysterectomy which removes the risk of uterine cancers.

In calculating the ACPDvD we use only first incident of the disease of interest as in [5], but we correct for the denominators in a different way using an assumption and some mathematics from the theory of competing risks. This second correction is detailed with precise mathematical notation in Fay et al [1]; here we give more heuristic arguments.

In the following let the disease of interest be "cancer". The ACPDvD between ages *x *and *y*, given alive and cancer-free at age *x*, may be written as the fraction,



To calculate the numerator, we integrate over the probability that the first cancer occurred at exactly age a. In math notation this probability is



where *f*_*c*_(*a*) is a probability function representing the probability that the first cancer occurred at exactly age a. One key result described in Fay et al [1] is that *f*_*c*_(*a*) can be written as the product of two functions,

*λ*_*c*_(*a*) = the probability that the first cancer occurred at exactly age a, given the individual is alive just before age *a*, and

*S*_*a*_(*a*-) = the probability that the individual is alive just before age *a*.

The function *λ*_*c*_(*a*) is known as a cause-specific hazard function, and it is estimated by some function of the age-specific rates, such as the piecewise constant model of Fay et al [1] or the MAJ model introduced in this paper (see Figure 1). Using standard results for continuous survival data, we can write *S*_*a*_(*a*-) as



where *λ*_*a*_(*u*) ( = the probability that the individual died at age *u*, given the individual is alive just before age *u*) is the usual hazard function. We estimate *λ*_*a*_(*u*) using some function of the age-specific rates. Thus, the numerator can be written as



**Figure 2:**
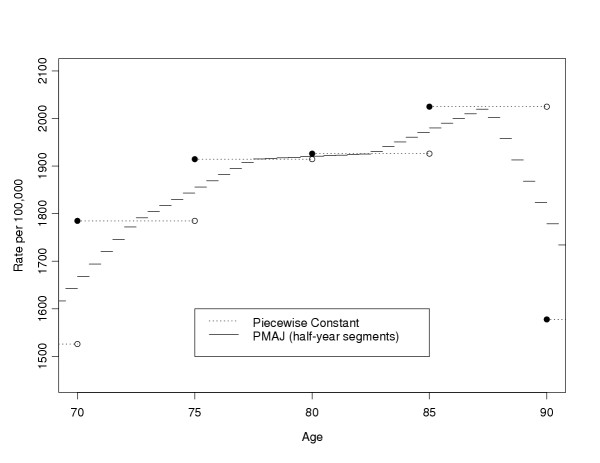
SEER 12 all invasive cancer incidence rates, 1998–2000, all races, both sexes: Piecewise constant and PMAJ methods.

If we use the MAJ for both hazard functions, then there is no closed form solution. To see why this is so, note that within the exponential, the integral of a piecewise linear function is the sum of a series of quadratic functions, and the overall integral has no closed form solution. This problem motivates the piecewise mid-age joinpoint (PMAJ) model, where we use a series of piecewise constant functions to approximate the MAJ model. Figure 2 gives the PMAJ model together with the piecewise constant model used by Fay et al [1] for 70 to 90 year olds from the SEER 12, 1998–2000 rates for all invasive (first) cancer incidence rates per person-years alive. Remember, although both Figure 1 and Figure 2 plot incidence rates, we additionally need similar rate functions for mortality rates to calculate the ACPDvD.

Now consider the denominator of the ACPDvD, the probability of being alive and cancer-free at age *x*, denoted . For reference, in Table 1 we give the notation. The only change from the notation in Fay et al [1] is that we use the subscript *a *to represent all causes of events instead of a blank subscript. For example, we let S*(*u*) = . Other notation in this paper is defined as it is introduced. Fay et al [1] assumed that the risk of death from other causes does not change if you have previously been diagnosed with cancer, then used the key result mentioned above together with some algebra and calculus to derive the denominator. Then the ACPDvD between the ages of *x *and *y *given alive and cancer-free just before age *x *is

**Table 1:** Notation

Random Variables and Parameters	
*T *= age at death	T* = age at first cancer or death before cancer
*J *= type of death	J* = type of event
(*J *= *d*) = death from cancer	(J* = *c*) = first cancer
(*J *= *o*) = death from other causes	(J* = *o*) = death before first cancer
*λ*_*c*_(*t*) = rate at *t *for first cancer given alive	= rate at *t *for first cancer given alive and cancer-free
*λ*_*o*_(*t*) = rate at *t *for death before cancer given alive	= rate at *t *for death before cancer given alive and cancer-free
*λ*_*d*_(*t*) = rate at *t *for death from cancer given alive	
*λ*_*a*_(*t*) = rate at *t *for death given alive	= rate at *t *for first cancer or death before first cancer given alive and cancer-free
	
Observations	

Within the age interval, [*a*_*i*_, *a*_*i*_+1), and within the calendar interval of interest we observe...	
*c*_*i *_= number of first cancer incident cases	= estimate of person-years alive associated with *j *= *c*, *d*, *o *(DevCan uses the sum of mid-year populations during the calendar interval of interest)
*d*_*i *_= number of cancer deaths	
*o*_*i *_= number of other deaths	



The details of the MAJ and the PMAJ models are given in the next two sections.

Readers only interested in the practical ramifications of the choice in models may skip to the examples and discussion section.

## Mid-age group joinpoint estimator

In Fay et al [1], the rates were estimated by a piecewise constant model. Here we use a mid-age group joinpoint (MAJ) model, where we draw lines connecting the midpoints of the intervals except the first and last interval. The first interval is constant until the midpoint, and the last interval is constant after a nominal "midpoint". This nominal "midpoint" is half the length of the previous age interval from the beginning of the last interval, and would be the midpoint if the last age interval was the same length as the previous interval.

We introduce new notation for breaking up the ages. Fay et al [1] used 0 = *a*_0 _<*a*_1 _< ··· <*a*_*k *_<*a*_*k*+1 _= ∞. Here we use a joinpoint model with joins at the midpoints (and nominal midpoint),



Let



(The indices start at -1 so that the index values for the rate estimators, , match up with the count notation of [1].) The MAJ estimator for the rate of event *j *(for *j *= c, d, or *o*) at *t*_*i *_(for *i *= 0,1,..., *k) *is



where *j*_*i *_is either *c*_*i*_, *d*_*i*_, or *o*_*i *_as defined in Table 1. (Note that , where  is the piecewise constant function used in [1]). We define  and . For *j *= *a*, MAJ estimator for the rate at *t*_*i *_is



Then for t ∈ [*t*_*i*_, *t*_*i*+1_) for *i *= 1,..., *k*, we define  as the point on the line defined by connecting the points (*t*_*i*_, ) and (*t*_*i*+1_, ). In other words,



Where



and



Thus, *α*_*j*,-1 _=  and *β*_*j*,-1 _= 0, and similarly by taking limits as *t*_*k*+1 _→ ∞ then *α*_*j*,*k *_=  and *β*_*j*,*k *_= 0.

Now  for *u *∈ [*t*_*i*_, *t*_*i*+1_) is



Note that (for ℓ = 0,1,..., *k*)



so that for *i *= 0,1,...,k,



Also notice that (when *u *< ∞)



Therefore when *u *∈ [*t*_*i*_*, t*_*i*+1_),



Let (*x, y*) be the estimator of *A*(*x, y*) using the MAJ model. The two integrals we need to estimate for (*x, y*) are of the type,



where in the numerator of (*x, y*) we need  (i.e., *j *= *c *and *h *= *a *in equation 7), and in the denominator of (*x, y*) we need . Suppose, without loss of generality, that *t *∈ [*t*_*i*_*,t*_*i*+1_), then



where *R*_*j*,*h*_(*t*_ℓ_, *v*) (for ℓ = - 1,0,1,2,..., *i *and *v *≤ *t*_ℓ+1_) is defined implicitly (see the Appendix). Then,



## Piecewise mid-age group joinpoint estimator

In the MAJ model we divided up the age line into *k *+ 2 intervals. Here we define those intervals in both the *t*_*i *_notation and the *a*_*i *_notation.



In the MAJ model the rates for the first and the last intervals are represented by lines with zero slope, and the rates for the *i*th interval (*i *= 1,...,*k*) for the *j*th rate type (*j *= *a*, *c*, *d*, *o*) is a line defined by connecting the points (*t*_*i*-1_, ) and (*t*_*i*_, ) (see equations 2 and 3 for definition of ). In the PMAJ model we divide the *i*th interval into *m*_*i *_equal sized intervals, and use a piecewise constant estimate on each of those *m*_*i *_intervals. One way to define *m*_*i *_is to chose *m*_*i *_so that each equal sized interval is 1/2 year long. In other words, *m*_*i *_= 2(*t*_*i *_- *t*_*i*-1_). This is the definition of *m*_*i *_that we use for the DevCan software (starting with version 5.0, see [3]), but all the following holds for arbitrary *m*_*i*_. In Figure 2 we show the PMAJ model with half-year intervals and the piecewise constant model for the US all invasive cancer mortality rates for ages 70 through 90 years.

Here are the details. Consider the *h*th (for *h *= 1,..., *m*_*i*_) of the *m*_*i *_intervals within interval *i *(for *i *= 1,...,*k*) for rate type *j *(for *j *= *a*, *c*, *d*, *o*). This interval is



For convenience we introduce new notation for the ends of this interval, let



so that *t*_*i*-1,0 _= *t*_*i*-1 _and  = *t*_*i*_. At the beginning of this interval the value of the rate is



(see equations 4 and 6 for definitions of *α*_*j*,*i*-1 _and *β*_*j*,*i*-1_). Similarly at the end of this interval the rate is



For the PMAJ model we simply assume a constant rate equal to the average of the beginning and the end values of the rate over this interval. In other words, under the PMAJ model for any *t *∈ [*t*_*i*-1,*h*-1_*,t*_*i*-1,*h*_) we estimate the rate with



Since the PMAJ model is a piecewise model, we can use Appendix A of [1] to express the estimator of age conditional probability of developing cancer. The only hard part is correctly defining the starting and ending of each piecewise interval. The ends of these intervals are



For convenience write these interval ends with only a single index as



where  and *m*_0 _= 1. In other words, *t*_-1 _= *τ*_0 _and for *i *= 0,1,..., *k*, then *t*_*i*_= *τ*_*g*_(*i*) and *t*_*i*,*h *_= *τ*_*g*_(*i*)+*h*, where .

Now we can follow very similar notation to Appendix A of [1]. We now repeat that Appendix with the modifications to notation required for the PMAJ model. Let the estimator of *A*(*x,y*) under the PMAJ model be denoted (*x,y*). Let *τ*_*i *_≤ *x *<*τ*_*i*+1 _and *τ*_*j *_<*y *≤ *τ*_*j*+1 _for *x *<*y,i *≤ *j*, and *j *≤ *M *+ 2. For convenience we regroup the ages after inserting group delimiters at *x *and *y*. Let the new delimiters be 0 = *b*_0_* ≤ b*_1 _≤ *b*_2 _≤ ··· ≤ *b*_*M*+3 _= ∞ where *b*_0 _= *τ*_0_,..., *b*_*i *_= *τ *_*i*_, *b*_*i*+1 _= *x, b*_*i*+2 _= *τ *_*i*+1_,..., *b*_*j*+1_= *τ *_*j*_, *b*_*j*+2 _= *y, b*_*j*+3 _= *τ*_*j*+1_,..., *b*_*M*+3 _= *τ*_*M*+1 _= ∞. We let



and similarly  and . In this notation, the probability of developing cancer by age *y *given survival until age *x *is *A*(*x, y*) = *A*(*b*_*i*+1_, *b*_*j*+2_), and under the PMAJ model we estimate it with



Because  or  may equal zero and *b*_ℓ+1 _may equal infinity, we let . These integrals are



where the case *λ *= 0 and *b*_ℓ+1 _= ∞ is one of the "impossible" hypothetical cohorts (see Section 3.1 of [1]). Thus, we obtain,



## Examples and discussion

In this section we explore several different methods for estimating the rate functions, all using the formula of Fay et al [1] (e.g., all using equation 1). This comparison explores the differences between the piecewise constant method proposed in Fay et al [1], the PMAJ method, and the MAJ method. A different comparison emphasizing differences between versions of the DevCan software is described in the supplemental material [see Additional file 1].

For all of the examples we use data from 1998–2000 [6]. The incidence data come from the Surveillance, Epidemiology, and End Results (SEER) program of the (U.S.) National Cancer Institute, and mortality data from the (U.S.) National Center for Health Statistics. We use the SEER 12 registries which cover about 14 percent of the U.S. population. We only use the mortality data covering the same area as the SEER 12 registries cover. Because the SEER 12 registries have complete coverage only back through 1992, we only look back in the database until 1992 to delete any incident case that had previously been diagnosed with the cancer of interest. These incident cases are deleted so that they are not counted when estimating the counts of first cancer incidence (the *c*_*i *_values). The mid-year population estimates (the *n*_*i *_values) come from the sum U.S. Census estimates of mid-year populations from 1998, 1999, and 2000 for the SEER 12 catchment areas for the appropriate sex group (e.g., males for prostate cancer).

In Table 2 we show the results for all invasive cancers and acute lymphocytic leukemia for both sexes, prostate cancer for males, and breast cancer for females. We see the PMAJ values approximate the MAJ values very well.

In conclusion, we have described several methods for estimating rates for input into a formula to calculate ACPDvD, and we have shown that the PMAJ method provides fast and reasonable estimators for the rates.

## Appendix: Calculation of *R *function

Recall that *R*_*j,h*_(*t*_ℓ_, *v*) represents an integral with 4 parameters. We can write it as



To simplify notation substitute let *t*_ℓ_ = *u *and *α*_*j*ℓ_ = *α*_*j*_*,β*_*j*ℓ _= *b*_*j*_,*α*_*h*ℓ _= *a*_*h*_, and *β*_*h*ℓ _= *b*_*h*_.

Thus,



### Case 1: *b*_*j *_= 0 and *b*_*h *_= 0

For our application, whenever *v *→ ∞ then *b*_*j *_= 0 and *b*_*h *_= 0, so this is an important special case.

When *b*_*j *_= 0 and *b*_*h *_= 0 and *a*_*h *_= 0 and we obtain



which goes to ∞ when *v *→ ∞.

When *b*_*j *_= 0 and *b*_*h *_= 0 and *a*_*h *_≠ 0 and we obtain



which goes to *a*_*j*_/*a*_*h *_when *v *→ ∞.

### Case 2: General Case with *v *< ∞

To calculate the integral, *R*(*u, v, a*_*j*_, *b*_*j*_, *a*_*h*_, *b*_*h*_) for finite *v*, we can use an adaptive use of Romberg's algorithm for numeric integration (we follow closely Lange [7], pp. 210–211).

Let



Divide the interval [*u, v*] into *n *equal subintervals of length (*v *- *u*)/*n*, and let



Then lim_*n*→∞ _*T*_*n *_= *R*(*u, v, a*_*j*_*, b*_*j*_, *a*_*h*_, *b*_*h*_).

A more accurate approximation uses Romberg's algorithm,



Let  be our estimate of *R*. The algorithm we use to calculate  is as follows:

1. Choose *n*.

2. Calculate *T*_*n*_.

3. Calculate *T*_2*n*_.

4. For i = 1 to *I*_*max *_do:

• If  then let  and stop.

• Otherwise calculate , and continue.

For example, one could use *n *= 100 and *δ *= 10^-5 ^and *I*_*max *_= 100.

## Supplementary Material

Additional File 1 Comparing the method of Wun, Merrill, and Feuer (1998) to the PMAJ method. We calculate lifetime risks of developing certain cancers for different race and sex combinations. For each lifetime risk we give the old method of Wun, Merrill, and Feuer [4], the PMAJ method, and the percent difference. In general, the two methods agree to within about 2 percent. Click here for file

## References

[B1] Fay MP, Pfeiffer R, Cronin KA, Le C, Feuer EJ (2003). Age-conditional probabilities of developing cancer. Statistics in Medicine.

[B2] Ries LAG, Eisner MP, Kosary CL, Hankey BF, Miller BA, Clegg L, Mariotto A, Fay MP, Feuer EJ, Edwards BK, eds (2003). SEER Cancer Statistics Review, 1975–2000. National Cancer Institute Bethesda, MD.

[B3] DevCan (2004). Probability of Developing or Dying of Cancer Software, Version 5.2. Statistical Research and Applications Branch, National Cancer Institute.

[B4] Wum L-M, Merrill RM, Feuer EJ (1998). Estimating lifetime and age-conditional probabilities of developing cancer. Lifetime Data Analysis.

[B5] Merrill RM, Feuer EJ (1996). Risk-adjusted cancer-incidence rates (United States). Cancer Causes and Control.

[B6] http://www.seer.cancer.gov.

[B7] Lange K (1999). Numerical Analysis for Statisticians.

