# Supplementary material for: Estimating age conditional probability of developing disease from surveillance data
Source: Popul Health Metr. 2004 Jul 27;2:6. doi: 10.1186/1478-7954-2-6 (PMC517510; doi:10.1186/1478-7954-2-6)
Supplement: Additional File 1 — Comparing the method of Wun, Merrill, and Feuer (1998) to the PMAJ method. We calculate lifetime risks of developing certain cancers for different race and sex combinations. For each lifetime risk we give the old method of Wun, Merrill, and Feuer [4], the PMAJ method, and the percent difference. In general, the two methods agree to within about 2 percent. [file 1478-7954-2-6-S1.pdf]

Comparing the method of Wun, Merrill, and Feuer (1998) to the PMAJ method

(This is supplemental material for: Fay, MP: **Estimating age conditional probability of developing disease from surveillance data**. *Population Health Metrics* 2004, http://www.pophealthmetrics.com/home/)

Since versions of the DevCan software prior to 5.0 used the method described in Wun, Merrill, and Feuer (1998), here we compare that method to the PMAJ method. Because some calculations were slightly off in versions 5.0 and 5.1, we use version 5.2 of DevCan (see DEVCAN,2004) with the corrected calculation. The bulk of the comparison has previously been done (see Fay et al. 2002). That comparison assumed the simple piecewise hazards models using the method described in Fay et al (2003). The only difference between the method described in Fay et al (2003) and that described in this paper is that in this paper we estimate the hazard functions with the PMAJ method.

In Table 3 (see pages 3-6) we recalculate Table I-15 from Ries et al. (2003) which gives lifetime risks of developing certain cancers for different race and sex combinations. We give the old method of Wun,Merrill, and Feuer (1998), the new method presented in this paper, and the percent differences. For the the Wun, Merrill, and Feuer (1998) method the age groups of the data must be in 5 year intervals except the last open ended interval. For the new method the data can be input with any age intervals, and for the example in Table 3 the first age interval is 1 year, the second is 4 years, and all subsequent intervals except the last are 5 years. Thus, the input data are slightly different for the two methods.

In general the two methods agree to within about 2 percent (see Table 3). The only cancer type with larger than about 2 percent in absolute difference is acute lymphocytic leukemia (ALL). For ALL the absolute percent differences are as large as 4.5 percent (for black males). One reason for that large absolute percent difference is the small absolute size of the ALL lifetime risk, so small absolute changes in risk translate to large absolute percentage changes. Another reason may be that ALL is a pediatric cancer, so the differences in the input data may be part of the cause of the differences.

For age conditional probabilities of developing cancers, the methods give similar answers. Although for very small probabilities the absolute percent difference can be very large, in those cases the absolute difference is small. For large probabilities where the absolute difference between the methods may be larger, the absolute percent difference is small.

References

DevCan: Probability of Developing or Dying of Cancer Software, Version 5.2. Statistical Research and Applications Branch, National Cancer Institute, http://srab.cancer.gov/devcan, 2004.

Fay, M.P., Pfeiffer, R., Cronin, K.A., Le, C. and Feuer, E.J. Comparison of Two Methods for Calculating Age-Conditional Probabilities of Developing Cancer. Technical Report #2002-01, Statistical Research and Applications Branch, National Cancer Institute 2002. (http://srab.cancer.gov/reports).

Fay, MP, Pfeiffer, R, Cronin, KA, Le, C,Feuer, EJ: **Age-conditional probabilities of developing cancer.** *Statistics in Medicine* 2003, **22**(11): 1837-1848.

Ries LAG, Eisner MP, Kosary CL, Hankey BF, Miller BA, Clegg L, Mariotto A, Fay MP, Feuer EJ, Edwards BK (eds): **SEER Cancer Statistics Review, 1975-2000,** National Cancer Institute. Bethesda, MD, http://seer.cancer.gov/csr/1975_2000, 2003.

Wun, L-M, Merrill, RM, and Feuer, EJ: **Estimating lifetime and age-conditional probabilities of developing cancer.** *Lifetime Data Analysis* 1998, **4:** 169-186.

Table 3: Lifetime Risk (percent) of Being Diagnosed with Cancer by Site, Race and Sex. 12 SEER Areas, 1998-2000. (Compare to Ries, et al. 2003, Table I-15). Each cell has 3 values: PMAJ method, Wun, Merrill, and Feuer (1998) method (WMF), and percent difference= $100(PMAJ - WMF)/WMF$.

	All Races		Whites		Blacks	
Site	Males	Females	Males	Females	Males	Females
All Invasive Sites	45.33	38.77	45.52	40.07	42.75	32.27
	44.88	38.65	45.05	39.90	42.47	32.38
	1.02	0.32	1.04	0.42	0.66	-0.35
Invasive and In Situ	46.54	42.08	46.82	43.53	43.13	34.52
	46.03	41.87	46.30	43.26	42.83	34.59
	1.11	0.50	1.14	0.61	0.70	-0.20
Oral Cavity and Pharynx	1.40	0.68	1.41	0.69	1.39	0.50
	1.41	0.68	1.42	0.69	1.41	0.50
	-0.72	-0.69	-0.66	-0.66	-1.22	-0.87
Esophagus	0.76	0.26	0.77	0.25	0.77	0.38
	0.76	0.26	0.77	0.25	0.79	0.39
	-0.87	-0.79	-0.81	-0.73	-1.34	-1.23
Stomach	1.26	0.80	1.09	0.66	1.33	1.04
	1.27	0.80	1.10	0.66	1.35	1.05
	-0.77	-0.74	-0.73	-0.70	-1.13	-0.99
Colon/Rectum	5.99	5.68	6.04	5.67	4.97	5.41
	6.01	5.71	6.07	5.70	5.02	5.46
	-0.47	-0.50	-0.40	-0.43	-0.98	-0.93
Invasive and In Situ	6.33	5.95	6.38	5.93	5.33	5.74
	6.36	5.98	6.40	5.96	5.38	5.79
	-0.43	-0.48	-0.37	-0.41	-0.93	-0.90
Liver and Intrahepatic Bile Duct	0.87	0.42	0.72	0.35	0.81	0.36
	0.87	0.42	0.73	0.36	0.82	0.37
	-0.81	-0.62	-0.74	-0.49	-1.33	-1.30
Pancreas	1.24	1.24	1.23	1.22	1.28	1.35
	1.25	1.25	1.24	1.23	1.30	1.36
	-0.88	-0.83	-0.83	-0.76	-1.26	-1.21
Larynx	0.65	0.16	0.65	0.17	0.87	0.24
	0.65	0.17	0.66	0.17	0.88	0.24
	-0.85	-0.76	-0.79	-0.72	-1.32	-1.16
Invasive and In Situ	0.70	0.18	0.71	0.18	0.89	0.25
	0.71	0.18	0.71	0.18	0.91	0.25
	-0.85	-0.76	-0.79	-0.71	-1.32	-1.16

Note: Invasive cancer only unless specified otherwise

Table 3: (continued) Lifetime Risk (percent) of Being Diagnosed with Cancer by Site, Race and Sex. 12 SEER Areas, 1998-2000. (Compare to Ries, et al. 2003, Table I-15). Each cell has 3 values: PMAJ method, Wun, Merrill, and Feuer (1998) method (WMF), and percent difference= $100(PMAJ - WMF)/WMF$.

	All Races		Whites		Blacks	
Site	Males	Females	Males	Females	Males	Females
Lung and Bronchus	7.78	5.80	7.77	6.09	8.35	5.40
	7.84	5.85	7.83	6.13	8.45	5.47
	-0.78	-0.75	-0.73	-0.68	-1.20	-1.16
Melanomas of Skin	1.83	1.24	2.17	1.48	0.11	0.08
	1.84	1.24	2.18	1.49	0.11	0.08
	-0.70	-0.61	-0.61	-0.54	-1.18	-0.07
Invasive and In Situ	2.89	1.97	3.39	2.33	0.13	0.12
	2.91	1.98	3.41	2.34	0.13	0.12
	-0.63	-0.60	-0.53	-0.52	-1.20	-0.42
Breast	0.11	13.55	0.12	14.31	0.14	10.20
	0.11	13.56	0.12	14.31	0.14	10.27
	-0.74	-0.08	-0.73	0.03	-0.92	-0.70
Invasive and In Situ	0.13	16.10	0.13	16.96	0.15	12.21
	0.13	16.09	0.13	16.92	0.15	12.28
	-0.75	0.08	-0.73	0.20	-0.94	-0.57
Cervix	NA	0.79	NA	0.76	NA	0.98
	NA	0.80	NA	0.76	NA	0.99
	NA	-0.60	NA	-0.51	NA	-1.08
Corpus and Uterus, NOS	NA	2.63	NA	2.83	NA	1.68
	NA	2.65	NA	2.85	NA	1.70
	NA	-0.62	NA	-0.53	NA	-1.14
Invasive and In Situ	NA	2.67	NA	2.88	NA	1.70
	NA	2.69	NA	2.90	NA	1.72
	NA	-0.61	NA	-0.53	NA	-1.13
Ovary	NA	1.72	NA	1.85	NA	1.11
	NA	1.73	NA	1.86	NA	1.12
	NA	-0.71	NA	-0.64	NA	-1.18
Prostate	17.34	NA	16.95	NA	20.54	NA
	17.22	NA	16.83	NA	20.39	NA
	0.69	NA	0.71	NA	0.77	NA
Testis	0.35	NA	0.42	NA	0.10	NA
	0.36	NA	0.42	NA	0.10	NA
	-0.35	NA	-0.34	NA	-0.48	NA

Note: Invasive cancer only unless specified otherwise

Table 3: (continued) Lifetime Risk (percent) of Being Diagnosed with Cancer by Site, Race and Sex. 12 SEER Areas, 1998-2000. (Compare to Ries, et al. 2003, Table I-15). Each cell has 3 values: PMAJ method, Wun, Merrill, and Feuer (1998) method (WMF), and percent difference= $100(PMAJ - WMF)/WMF$.

	All Races		Whites		Blacks	
Site	Males	Females	Males	Females	Males	Females
Urinary Bladder (In Situ and Inv)	3.53	1.14	3.93	1.22	1.43	0.78
	3.55	1.14	3.94	1.23	1.45	0.79
	-0.54	-0.77	-0.44	-0.69	-1.15	-1.21
Kidney and Renal Pelvis	1.46	0.87	1.54	0.91	1.24	0.86
	1.48	0.88	1.55	0.92	1.26	0.87
	-0.78	-0.75	-0.70	-0.73	-1.33	-0.77
Brain and Other Nerv Sys	0.67	0.53	0.75	0.59	0.32	0.30
	0.67	0.53	0.75	0.59	0.33	0.31
	-0.93	-0.70	-0.82	-0.69	-1.55	-1.37
Thyroid	0.30	0.85	0.32	0.87	0.14	0.46
	0.30	0.85	0.32	0.88	0.15	0.47
	-0.74	-0.57	-0.66	-0.49	-1.30	-1.08
Hodgkin's Disease	0.23	0.20	0.26	0.22	0.19	0.15
	0.24	0.20	0.26	0.22	0.19	0.15
	-0.78	-0.59	-0.72	-0.52	-1.22	-0.95
Non-Hodgkin's Lymphomas	2.13	1.80	2.26	1.91	1.18	1.05
	2.15	1.81	2.28	1.93	1.19	1.06
	-0.78	-0.77	-0.71	-0.70	-1.26	-1.13
Myeloma	0.66	0.54	0.65	0.49	0.89	0.93
	0.67	0.54	0.66	0.50	0.91	0.94
	-0.88	-0.87	-0.82	-0.81	-1.33	-1.27
Leukemias	1.45	1.03	1.55	1.09	0.90	0.74
	1.47	1.04	1.56	1.10	0.91	0.75
	-0.90	-0.89	-0.86	-0.88	-1.40	-1.03
Acute Lymphocytic Leukemia	0.12	0.10	0.13	0.12	0.06	0.06
	0.12	0.11	0.13	0.12	0.07	0.06
	-3.39	-2.46	-3.51	-2.87	-3.85	0.88
Chronic Lymphocytic Leukemia	0.47	0.29	0.51	0.31	0.30	0.19
	0.47	0.29	0.52	0.32	0.30	0.20
	-0.77	-0.74	-0.69	-0.66	-1.32	-1.25
Acute Myeloid Leukemia	0.45	0.35	0.47	0.36	0.29	0.28
	0.45	0.36	0.47	0.37	0.29	0.28
	-0.59	-0.76	-0.56	-0.70	-0.98	-1.26

Note: Invasive cancer only unless specified otherwise

Table 3: (continued) Lifetime Risk (percent) of Being Diagnosed with Cancer by Site, Race and Sex. 12 SEER Areas, 1998-2000. (Compare to Ries, et al. 2003, Table I-15). Each cell has 3 values: PMAJ method, Wun, Merrill, and Feuer (1998) method (WMF), and percent difference= $100(PMAJ - WMF)/WMF$.

	All Races		Whites		Blacks	
Site	Males	Females	Males	Females	Males	Females
Chronic Myeloid Leukemia	0.19	0.14	0.20	0.14	0.14	0.10
	0.20	0.14	0.20	0.14	0.14	0.10
	-0.76	-0.83	-0.68	-0.78	-1.39	-1.05

Note: Invasive cancer only unless specified otherwise
